# Mapping Disease Transmission Risk of Nipah Virus in South and Southeast Asia

**DOI:** 10.3390/tropicalmed3020057

**Published:** 2018-05-30

**Authors:** Mark A. Deka, Niaz Morshed

**Affiliations:** Department of Geography, Texas State University, 601 University Drive, San Marcos, TX 78666, USA; m_m617@txstate.edu

**Keywords:** Nipah virus, ENMeval, BIOMOD2, risk mapping, ecological niche modeling, disease biogeography, infectious disease cartography

## Abstract

Since 1998, Nipah virus (NiV) (genus: *Henipavirus*; family: Paramyxoviridae), an often-fatal and highly virulent zoonotic pathogen, has caused sporadic outbreak events. Fruit bats from the genus *Pteropus* are the wildlife reservoirs and have a broad distribution throughout South and Southeast Asia, and East Africa. Understanding the disease biogeography of NiV is critical to comprehending the potential geographic distribution of this dangerous zoonosis. This study implemented the R packages ENMeval and BIOMOD2 as a means of modeling regional disease transmission risk and additionally measured niche similarity between the reservoir *Pteropus* and the ecological characteristics of outbreak localities with the Schoener’s D index and *I* statistic. Results indicate a relatively high degree of niche overlap between models in geographic and environmental space (D statistic, 0.64; and *I* statistic, 0.89), and a potential geographic distribution encompassing 19% (2,963,178 km^2^) of South and Southeast Asia. This study should contribute to current and future efforts to understand the critical ecological contributors and geography of NiV. Furthermore, this study can be used as a geospatial guide to identify areas of high disease transmission risk and to inform national public health surveillance programs.

## 1. Introduction

In a 2005 review on emerging and reemerging infectious agents, of the 1407 human pathogens, 816 (58%) were classified as zoonotic in origin [1]. In recent decades, zoonotic pathogens have induced considerable stress and anxiety in a broad range of societies worldwide. The emergence of Nipah virus (NiV) in Peninsular Malaysia in September 1998 was the second in a series of spillover events. The first, starting in September 1994, was an outbreak of Hendra virus (HeV) in Brisbane, Australia [2,3,4,5]. Nipah and Hendra viruses are members of the family Paramyxoviridae (genus: *Henipavirus*), each can potentially cause fatal disease in human and animal hosts [6]. Nipah virus takes its name from the village of Kampung Sungai Nipah in Malaysia, where the virus was isolated from pigs presenting neurological and respiratory symptoms [7,8]. NiV-infected pigs developed a unique clinical condition called ‘barking pig syndrome’ [9]. The first human cases in Malaysia (Perak, Negri Sembilan, and Selangor states) and Singapore were reported amongst abattoir workers.

The Malaysia epidemic resulted in 265 cases of acute encephalitis with 109 deaths and the culling of 1.1 million pigs [10,11]. Since 1998, Malaysia and Singapore have not documented human cases; however, human disease has been continuously reported in Bangladesh since 2001, with mortality rates estimated at approximately 70% [12]. Subsequently, NiV has emerged as a significant public health threat in Bangladesh and India [13]. Unlike the initial outbreak, in which pigs were the primary host, the role of bat reservoirs in human infection has been substantiated [14]. The geography of NiV in Bangladesh, exhibits characteristics of clustering, particularly in the Dhaka, Khulna, Rajshahi, and Rangpur divisions. Date palm monoculture and the geographic distribution of transmission events since 2001 display strong spatial dependency [4,15,16]. Bats (order: Chiroptera) of the family Pteropodidae, genus *Pteropus* (flying foxes) are the presumed wildlife reservoir of NiV [17]. *Pteropus giganteus* or the Indian flying fox is the largest frugivorous bat species in Bangladesh and is of key interest as the zoonotic reservoir of Nipah virus. *Pteropus giganteus* is further associated with harboring at least 55 recently-discovered viruses [18]. The asymptomatic nature of NiV in bats suggests that the virus had evolved alongside *Pteropus* bats for centuries, and more than likely this adaptation has been responsible for human exposure long before the virus was first reported in 1998 [19,20,21]. Biological traits making bats well-suited for hosting a variety of microorganisms include their long lifespans, which facilitate viral persistence [22] and their ability for flight. Long-distance travel is prevalent; in fact, the grey-headed flying fox (*Pteropus poliocephalus*) expands its range by up to 600 km during long-distance travel between roosting sites [23,24,25]. Regionally, 330 species of bats are endemic to Southeast Asia, which accounts for 25% of the world’s overall bat diversity [26]. The genus *Pteropus* alone features 60 species of bat with broad geographic distributions extending to the east coast of Africa, the Philippines, Indonesia, New Guinea, and much of the Indian sub-continent [6].

NiV is classified as a high-priority agent of biological warfare by the Centers for Disease Control and Prevention [27] and causes severe respiratory and febrile encephalitic illness in humans after an incubation period between 4–45 days [28]. Symptoms range from fever, headache, myalgia, disorientation, seizure, vomiting and coma, with a case mortality rate ranging from 40–70% [29,30,31]. Viruses from the genus *Henipavirus* can infect a wide range of mammalian species and outside of NiV and HeV, include recently discovered Cedar (CedV), Kumasi (KV), and Mojiang virus (MojV). The primary risk factors for contracting NiV in Bangladesh and eastern India are tied to the consumption of raw date palm sap, contaminated with the urine or saliva of *Pteropus* bats, direct contact with infected humans, and hunting bats for bushmeat [4,15,18,32,33,34,35]. Studies using infrared cameras have shown that *Pteropus* bats visit date palm trees at night and contaminate sap by licking and urinating in the collection pots [36]. Those who contracted the disease following the consumption of raw or fermented date palm sap had a higher case fatality rate compared to those individuals who developed illness through direct exposure to an infected human [37]. Reports from India confirm that *Pteropus* bats are hunted for both food and medicine and are used as treatments in rural areas for asthma and chronic pain [38]. Pathogen spillover begins when a viral agent jumps from an animal reservoir to humans with minimal subsequent human–human transmission [39]. During these repeated exposures, a phenomenon known as ‘viral chatter’, transformations may develop making it more likely that the pathogen will spread to humans [39]. Spillover is a critical antecedent and serves as a significant upstream source for human–human transmission [40]. According to Plowright et al. [41], a series of interconnected conditions are necessary for the facilitation of spillover events from bats. Bats, of course, must be present in the environment and must be *infected* and actively *shedding* the pathogen. Outside of the reservoir, the virus must *survive* in the environment and have access to a recipient host in sufficient quantities to cause illness.

Previous efforts to model and identify the pertinent ecological contributors and geography of NiV are limited and vary considerably by the scale of analysis. Peterson [42] and Hahn et al. [43] developed ecological niche models for Bangladesh, based on human occurrences and *Pteropus* roosting sites. While Walsh [44] took a broad scale approach across South and Southeast Asia using an inhomogeneous Poisson model. Disease modeling and risk mapping contribute to a better understanding of ecology, epidemiology, and disease biogeography, while providing an objective basis for public policy formulation [45]. Disease biogeography and infectious disease cartography (infectious disease mapping) are emerging fields of study, merging quantitative mapping with the study of infectious disease, vectors, reservoirs, and susceptible hosts [46,47]. Disease biogeography shares linkages with epidemiology and ecology through the application of analytical toolsets to study the distribution of epidemic events [46]. Infectious disease cartography similarly applies analytical techniques as a means of quantifying disease transmission risk through deterministic [48], statistical [49], and geostatistical modeling [26]. Together these frameworks provide evidence-based policymaking for public health officials focused on mitigating the effects of infectious agents in human and animal populations.

In recent decades, South and Southeast Asia have become the location of emerging and re-emerging infectious diseases, due to a combination of inadequate public health systems, rapidly expanding human populations, and an abundance of potential wildlife hosts [50]. Future efforts to describe the geographic variation in the disease transmission risk of NiV infection regionally would benefit from an understanding of the disease biogeography of the primary host *Pteropus*, and of the environmental characteristics of NiV transmission localities. The primary aim of this study was to: (1) provide contemporary disease maps that delineate the most significant risk for NiV in South and Southeast Asia; (2) identify those abiotic and biotic features associated with increased risk; and (3) to evaluate geostatistical models to ascertain varying degrees of model overlap in geographic and environmental space. Because of the imminent public health threat associated with NiV, the need for detailed risk maps is necessary to improve disease surveillance, control systems, and to further, minimize human mortality, long-term morbidity, economic distress, and spillover events.

## 2. Materials and Methods

### 2.1. Study Area

**Figure 1:**
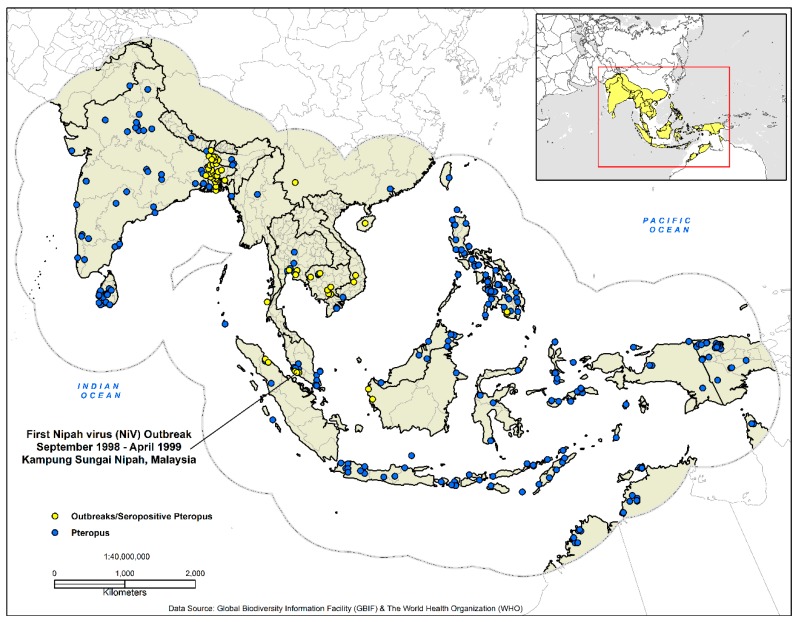
Presence-only data for the development of the disease transmission risk models. The dashed outline in grey demarcates the analysis area at 770 km, which represents the accessibility or potential movement (**M**) as defined by the ‘BAM’ framework.

This study covered 15,595,674 km^2^ (17.55° S to 38.55° N, 64–146° E) (Figure 1) and included the countries of Afghanistan, Australia, Bangladesh, Brunei, Cambodia, China, Hong Kong, India, Indonesia, Laos, Macau, Malaysia, Maldives, Myanmar, Nepal, Pakistan, Papua New Guinea, the Philippines, Singapore, Sri Lanka, Taiwan, Thailand, Timor-Leste, and Vietnam. An essential element in the development of ecological niche models are hypotheses of the areas (M) that are potentially accessible to the species; a theory incorporated into the three-factor conceptual ‘BAM’ framework (biotic, abiotic, movement) [51,52]. With this in mind, we delineated our study area as recommended by Peterson and Samy [53]. South and Southeast Asia are home to an estimated 2,884,289,620 people or 39% of the world population and produces a vast economic output estimated annually at $9.107 trillion (GDP—nominal) [54,55,56] (Figure 1).

### 2.2. Data Collection

Biosurveillance data for Nipah outbreaks and seropositive *Pteropus* bats (*n* = 51) (1998–2014) were acquired from the Institute of Epidemiology, Disease Control and Research (IEDCR) (Dhaka, Bangladesh) [57], the World Health Organization [16], and a variety of diverse literature sources [8,13,58,59,60,61,62,63]. Vector-borne and zoonotic agents are spatially dependent on the geographic distribution of both their vectors and hosts. To discern the risk of disease transmission, it is imperative to identify the geographic distribution of the host species [64,65,66]. NiV has been documented in bats from the genus *Pteropus*; we utilized The Global Biodiversity Information Facility (GBIF) (GBIF.org (18 November 2017) GBIF Occurrence Download https://doi.org/10.15468/dl.utprbq) to identify the geographic distribution of *Pteropus* sightings and specimens (*n* = 918). Finally, both datasets were georeferenced in ArcGIS 10.5.1 with a geographic coordinate system [67].

### 2.3. Model Covariates

With the aim of identifying abiotic and biotic features and their association with *Pteropus* hosts and human transmission events, a variety of high-resolution geospatial datasets were applied (Table 1). In acquiring climate data, the widely-cited WorldClim Global Climate database was not used [54], due to recently identified limitations [55]. As an alternative, nineteen-MERRAclim (Modern-Era Retrospective Analysis for Research and Applications) bioclimatic variables from 2000–2010 [56] (2.5 arc-minutes) were incorporated. MERRAclim coverages are constructed through homogenous hourly temperature and humidity data collected by NASA (National Aeronautics and Space Administration) satellite reanalysis of 28 data products and represent nineteen bioclimatic variables [68]. The global distribution of land cover, enhanced vegetation index (EVI), land surface temperature (LST) and elevation at a resolution of 1-km coverages were acquired from the Land Processes Distributed Active Archive Center (LP-DAAC) (https://lpdaac.usgs.gov/), and the NASA Jet Propulsion Laboratory—California Institute of Technology (https://www2.jpl.nasa.gov/srtm/).

Because of the implications of anthropogenic activities on the emergence of zoonotic and vector-borne diseases, gross forest cover loss (2000–2016) was acquired at a 30-m resolution from the Global Forest Watch (GFW) (http://earthenginepartners.appspot.com/science-2013-global-forest/download_v1.4.html) [69]; and high resolution human population density grids (2015) at a 1-km resolution from the GeoData Institute (www.worldpop.org.uk). Gross forest loss is a change from forest to non-forest, or a stand-replacement disturbance [69]. Additionally, incorporated were data depicting the presence of tree plantations in Southeast Asia at a 1-km resolution. This dataset, created by Transparent World (http://www.transparentworld.ru/en/), with direct support from the Global Forest Watch (GFW), features geospatial data representing a variety of plantations ranging from oil palm, fruit, coconut palm, cacao, and wood fiber/timber. All forms of domesticated zoonotic reservoirs including cattle, goats, sheep, and pigs were considered. These considerations were justified because of the amplification of the virus by pigs, and the results of a recent study that identified NiV antibodies circulating in cattle and goat populations in Bangladesh [70]. The global distribution was represented with the Gridded Livestock of the World products via the Food and Agricultural Organization of the United Nations (FAO) (1-km resolution) (http://www.livestock.geo-wiki.org). All model covariates were preprocessed at a 1-km resolution with identical cell sizes and spatial reference systems. To define potential biologically-relevant variables and to guard against multi-collinearity and over-parameterization, we performed a measure of variance inflation factors (VIF) [71] on all 34-model covariates. In total, seventeen covariates were excluded before the model calibration phase, and these include one land cover covariate (broadleaf biomes), the density of goats, and all MERRAclim rasters other than temperature seasonality, the mean temperature of driest quarter, the mean temperature of warmest quarter, and precipitation of warmest quarter.

### 2.4. Model Calibration

To quantify disease transmission risk, the R 3.4.1 [72] packages ENMeval [73] and BIOMOD2 [74] were used for the analysis. The first step in our study was to test both presence-only datasets in ENMeval or *f*MaxEnt (fine-tuned MaxEnt) to determine the correct feature class transformations (FC) and *ß* regularization multipliers (RMvalues). ENMeval facilitates the construction of ENM’s based on the presence-only method, MaxEnt (v. 3.3.4k) [75]. The ENMeval package calculates evaluation metrics as an alternative to arbitrarily assigning model parameters. ENMeval allows the user to conduct spatially independent evaluations and partition data through the implementation of *k*-fold cross-validation, and further determines model fit based on the Akaike Information Criterion (AIC) and provides six options for data partitioning. Of the six, the *n* − 1 jackknife method was used. Models in R were built with a range of *ß* regularization multipliers (RM values) from 0.5 to 4.0 (increments of 0.5) to test for and prevent model over-complexity and overfitting. All possible feature class (FC) transformations (L, LQ, H, LQH, LGHP, and LQHPT), those being linear (L), quadratic (Q), hinge (H), product (P), and threshold (T), were used resulting in 500 individual model replicates. Modeling methods such as MaxEnt display some of the highest predictive ability in identifying favorable suitability outside of known occurrence locations [76]. The principle of maximum entropy estimation, first described by [77], from a theoretical perspective is a Bayes estimation method [78]. MaxEnt predicts the relative occurrence rate (ROR) in each cell based on the environmental characteristics at each PO location [79].

The second step involved creating a consensus or ensemble model (RM) using the BIOMOD2 package. BIOMOD2 gives the user the option of implementing machine learning, classification, regression and surface range envelope techniques using presence/absence data and environmental covariates. The central basis for ensemble forecasting operates on the premises that by using several modeling methods that a measure of central tendency (mean or median) can be quantified, and thus a more reliable prediction can be made [80]. Eleven algorithms were applied in this study: generalized linear models (GLM) [81], generalized additive models (GAM) [81], generalized boosting model (GBM) [82], classification tree analysis (CTA) [83], artificial neural networks (ANN) [84], surface range envelop (SRE) [85], flexible discriminant analysis (FDA) [86], multiple adaptive regression splines (MARS) [87], random forest (RF) [88], maximum entropy (Maxent. Phillips) [75], and low-memory multinomial logistic regression (Maxent. Tsuruoka) [89]. Other than the maximum entropy (Maxent. Phillips) technique, which included the ENMeval defined feature-class settings and *ß* regularization multipliers, the remaining algorithms featured the BIOMOD2 default model settings. Model parameters specified a true skill statistic (TSS) evaluation metric quality threshold of <0.5, a measure of weighted mean probability and five evaluation runs. Variable importance in BIOMOD2 is based on the decrease in accuracy and through correlating, the fitted data of the full models with the predictor values that are randomly permuted [80].

**Figure 2:**
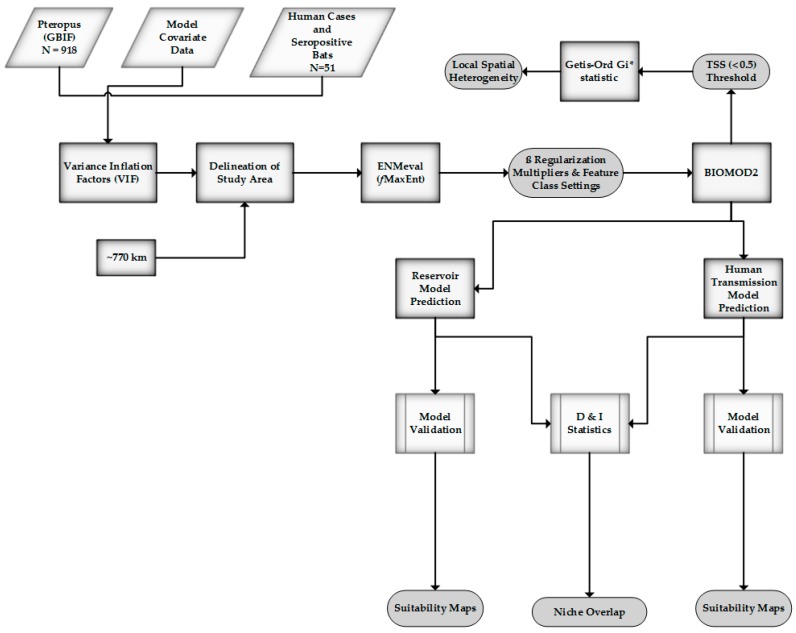
Method overview.

Models were built using 70% of the occurrences data; the remaining 30% was withheld for validation. Model evaluation featured three statistical metrics: receiver-operating curve (ROC), Cohen’s Kappa (KAPPA), and the true skill statistic (TSS). The ROC (AUC) is derived from a comparison of the null model with a random predictive AUC value equal to 0.50, models with AUC values >0.75 have the potential of being quite useful [90]. Cohen’s Kappa compares observed accuracy with a measure of expected accuracy (random chance) and is regarded as a robust measure of agreement calculation [91]. The TSS similarly measures model performance by considering omission and commission errors and ranges from −1 (random) to +1 (perfect model agreement) [92]. An inherent advantage of the TSS is that it retains the strong properties of KAPPA while not experiencing issues with sensitivity [93]. An overview of the methods can be found below (Figure 2).

### 2.5. Geospatial Analysis

Following the exportation of the ensemble models, binary maps were produced from the selected TSS quality threshold values (<0.5) specifying presence and absence. The binary maps were merged based on intersecting grid cells using weighted overlay analysis [ESRI] and projected (PCS) to calculate the suitable land area in km^2^. To measure and analyze the existence and evidence of local spatial heterogeneity and clustering in our threshold values, the Getis-Ord Gi* statistic (global indicator of spatial association) [94,95] was applied through ArcGIS 10.5.1 spatial statistics tools [67]. Spatial heterogeneity refers to the uneven distribution of various phenomena in space; this can be attributed to either local spatial heterogeneity or stratified spatial heterogeneity [96]. The Gi* statistic (spatial conceptualization: inverse distance) evaluated whether these features were clustered, dispersed, or random. The tool identifies statistically-significant spatial clusters of high values (high spots) or low values (cold spots). For a hot spot to be classified as statistically significant, a feature with a high value must be surrounded by elements with similar values. The Getis-Ord Gi* is as follows [94]:

(1) $$G_{i}^{*}=\;\frac{\sum_{j=1}^{n}\omega i,jX_{i-\bar{X}}\sum_{j=1}^{n}\omega i,j}{S\sqrt{\left[n\sum_{j=1}^{n}\omega_{i,j}^{2}-\;{\left(\sum_{j=1}^{n}\omega i,j\right)}^{2}\right]}}$$

where $x_{i}$ is the attribute value for the j features, ωi,j represents the spatial weight between feature i and j, and *n* is equal to the total number of features as:

(2) $$\bar{X}=\frac{\sum_{j=1}^{n}x_{j}}{n}$$

(3) $$S=\;\sqrt{\frac{\sum_{j=1}^{n}x_{j}^{2}}{n}}-\left(\bar{X}\right)2$$

### 2.6. Measuring Niche Overlap

The overlapping distribution of reservoir and recipient hosts delineates geographies where the recipient hosts are at an increased risk for infection [41]. To measure similarities between model surfaces and to evaluate the niche conservatism in environmental ($\Delta_{env})$, and geographic space ($\Delta_{geo})$, two statistical measures—Schoener’s *D* index [97] and the *I* statistic from Hellinger distance [66]—were employed. Geographic space in this analysis represents the **M** hypotheses of accessibility, while the second facet, ecological space, or ‘natural space’ defines the environment separate from human activity or the interdependence between physical and living constituents [98]. Both methods were incorporated in ENMTools [66]. These statistics employ metrics ranging from 0 (no overlap) to 1 (>0.6 significant overlap). The *I* and *D* statistics determine overlap by calculating the difference between models in the suitability scores within each grid cell [66]. Niche overlap is calculated using Schoener’s *D* with the following formula:

(4) $$D\left(p_{x,}p_{y,}\right)=1-\;\frac{1}{2\;}\underset{n=i}{\sum }\left|P_{x,i-}P_{y,i-}\right|$$

where $P_{x,i}$ and $P_{y,i}$ signify the probability assigned by the ENM to grid cell *i* for species *x* and *y* [99]. Schoener’s *D* applied to $P_{x,i}$ values establish the degree of geographic overlap between the *Pteropus* reservoir distribution and human transmission models. The *I* statistic on the contrary measures true suitability without prior biological assumptions $P_{x,i}$ and is defined as [66,99]:

(5) $$I\left(P_{x},P_{y}\right)=1-\;\frac{1}{2}\;H\left(P_{x,}P_{y}\right)$$

Hellinger distance or *H* is defined as:

(6) $$H\left(P_{x},P_{y}\right)=\;\sqrt{\underset{i}{\sum }\left(\sqrt{P_{x,i}}-\;\sqrt{P_{y,i}}\right)}$$

## 3. Results

After tuning the reservoir PO data and environmental coverages in fMaxEnt, the most robust model performance was achieved with all feature classes (LQHPT) and a regularization multiplier of 4.0 (*ß*). The top-performing techniques in BIOMOD2 were the generalized linear model (GLM), generalized boosting model (GBM), flexible discriminant analysis (FDA), multiple adaptive regression splines (MARS) and random forest (RF) algorithms. The maximum entropy and low-memory multinomial logistic regression (Maxent. Tsuruoka) methods performed poorly and were excluded from the final ensemble output. Model evaluation values between ROC, KAPPA, and TSS were acceptable and ranged from 0.606 to 0.897 (Table 2). The most accurate model when comparing ROC, KAPPA and TSS metrics was the random forest algorithm.

Highly favorable conditions are found in equatorial regions extending past the Wallace Line (faunal line) to New Guinea, the Philippines, west through Southern Vietnam, Cambodia, Thailand, a substantial proportion of the Indian sub-continent, southwestern Pakistan, southern China and northern Australia. Population density (importance: 0.286), mean temperature of the driest quarter (importance: 0.252), precipitation of the warmest quarter (importance: 0.143), land surface temperature (LST) (importance: 0.124), and temperature seasonality (importance: 0.117) were critical environmental predictors (Table 3).

The best performance and ideal settings for the human transmission model was a regularization multiplier of 1 (*ß*), and a linear (L) only feature class setting. The top-performing techniques were the generalized linear model (GLM), generalized boosting model (GBM), flexible discriminant analysis (FDA), multiple adaptive regression splines (MARS), random forest (RF), maximum entropy (Maxent. Phillips), and low-memory multinomial logistic regression (Maxent. Tsuruoka)algorithms (Table 4). Cattle density (importance: 0.509), temperature seasonality (importance: 0.201), elevation (importance: 0.115), land surface temperature (LST) (importance: 0.105), population density (importance: 0.103), the mean temperature of the driest quarter (importance: 0.097), and the mean temperature of the warmest quarter (importance: 0.088) were significant model contributors (Table 5). The most accurate models were the machine learning techniques: random forest and the generalized boosted model. Geographically, a high probability of occurrence was predicted on coastal and highly populated areas in southern China, the Mekong Delta, Peninsular Malaysia, Java and Sumatra, Indonesia, the Irrawaddy Delta, India and Sri Lanka.

**Figure 3:**
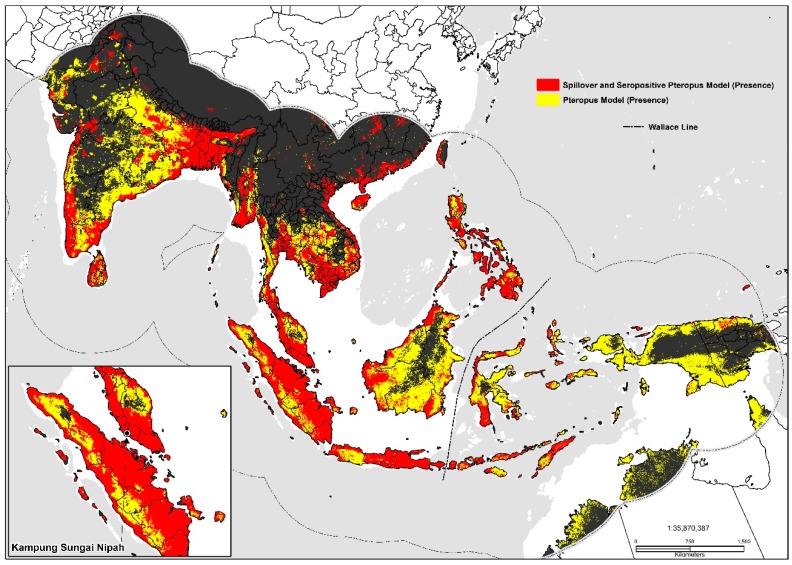
Predicted geographic distribution in South and Southeast Asia.

Large inland swaths of high suitability are identified throughout the southern and northwestern Indo-Gangetic Plain, southern Pakistan, Bangladesh, and the Brahmaputra Basin. The predicted distribution is additionally related to the density of sheep (0.06), pigs (0.06), mosaic vegetation (0.051), and the presence of tree plantations (0.032). Combined the geographic distribution between models display’s high suitability on coastal stretches of southern China, India, inland portions of the Deccan Plateau, southern Nepal, the Indo-Gangetic Plain, Indus Basin, Greater Mekong Subregion, Taiwan, the Philippines, and Indonesia (Figure 3). Maps displaying risk at the country level for selected areas were also produced (please see Supplementary Materials: S1, S2, S3).

### Geospatial and Niche Overlap Analysis

**Figure 4:**
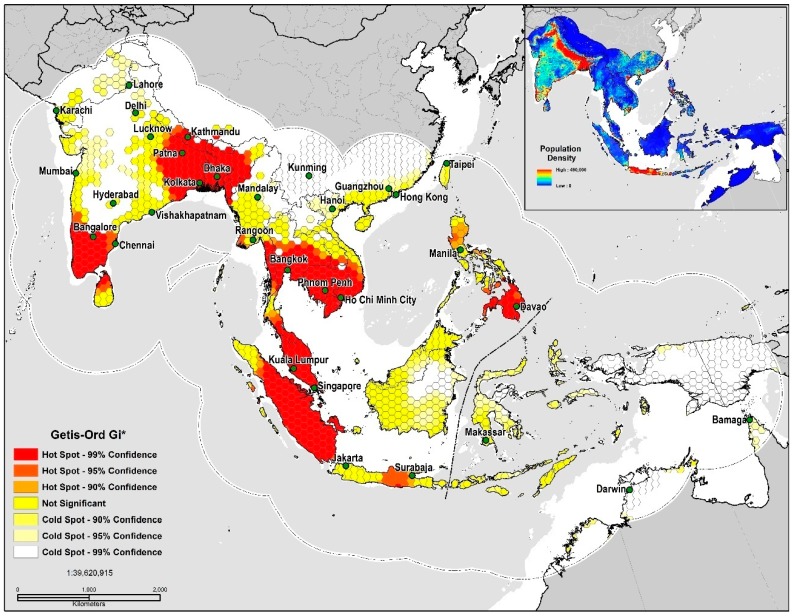
TSS quality threshold (<0.5) Getis-Ord Gi* Hot Spot Analysis.

In measuring spatial autocorrelation based on the TSS quality threshold (<0.5) values, the presence of high positive local spatial heterogeneity is strong in the 90–99% CL (GiZscore: 5.33; GiPvalue: 0.0052) and exhibits clustering and spatial dependency at eight locations, the southeastern Indo-Gangetic Plain, Indonesia, Peninsular Malaysia, the Greater Mekong Subregion, southern India, northern Sri Lanka, the Irrawaddy Delta, and the Philippines (Figure 4). The land area deemed as high risk for disease transmission totaled 2,963,178 km^2^ or 19% of the study area. Niche equivalency tests of the *D* metric equaled a value at 0.64 indicating a relatively high degree of overlap between models. The *I* statistic when solely based on the probability distribution was much higher at 0.89; inferring that a very high level of similarities exists in environmental ($\Delta_{env})\;$ and geographic space ($\Delta_{geo})$. In sum, our results indicate that both models share strong similarities in their ecological niches.

## 4. Discussion

This study mapped the potential disease transmission risk of Nipah virus (NiV) in South and Southeast Asia. This analysis used information from spillover events and *Pteropus* bats to delineate the likely niche of NiV. By accounting for the geographic distribution of both human transmission events and the reservoir species, significant ecological contributors governing disease transmission risk and the disease biogeography of Nipah virus are revealed. Our investigation has demonstrated that environmental suitability in South and Southeast Asia is extensive. These findings suggest that, when covariates are ranked collectively between models that population density, cattle density, mean temperature of the driest quarter, temperature seasonality, land surface temperature (LST), elevation, mean temperature and precipitation of the warmest quarter, mosaic vegetation, pig density, enhanced vegetation index (EVI), sheep density and tree plantations are the most significant contributors. The models presented in this study display a very high degree of visual agreement. Most of the areas predicted as highly suitable (presence) coincide with areas that have documented the presence of *Pteropus* bats and human cases; however, broad regions without reported human infection were also predicted (Table 6). Our analyses further suggested that the spatial clustering of evaluation thresholds (<0.5) is concentrated primarily in the densely populated western half of the study area.

Our models predicted a high degree of environmental suitability in vast areas of the Indian sub-continent, Indonesia, Southeast Asia, Pakistan, southern China, northern Australia, and the Philippines. In comparing our results to broad-scale analysis of NiV by Walsh [4], which is the most appropriate for comparison, our models predicted an increase in disease transmission-risk over stretches of India, Pakistan, Borneo, and portions of western New Guinea. Measures of niche overlap in environmental ($\Delta_{env})$, and geographic space ($\Delta_{geo})$ indicate that a relative to very high correlation exists between the reservoir and human transmission models. To our knowledge, this research is the first to measure niche overlap between the wildlife reservoir of NiV, spillover events and seropositive bats, a finding that accounts for the persistence of the virus at the reservoir and landscape level. Moving beyond niche overlap, the influence of scale must be accounted for. At the coarsest or finest scales, the manifestation of pathogen exposure varies considerably, and is driven by numerous human, economic and social structures; as well as the phylogenetic closeness to the reservoir host species [100]. The implications of measuring such correlations have a high degree of importance to public health and disease transmission ecology, since the eco-epidemiology of NiV is both sylvatic and synanthropic [101,102].

**Figure 5:**
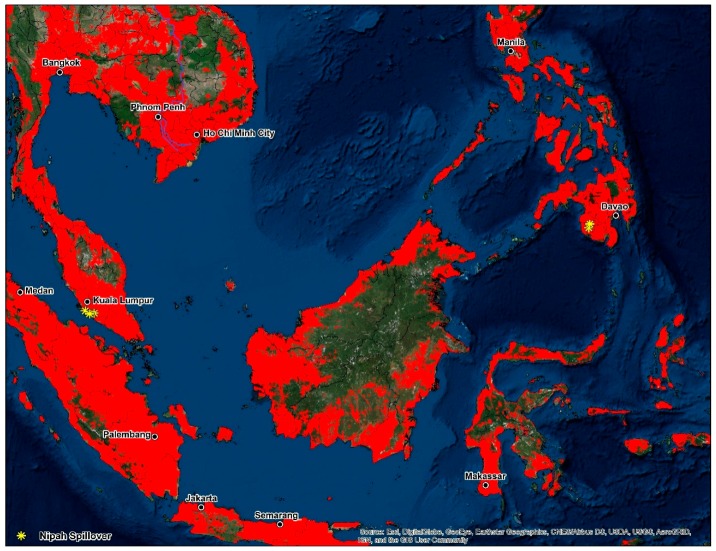
Disease transmission risk—Southeast Asia.

Models developed in this study delineated large areas of high disease transmission risk through much of South and Southeast Asia, especially in the proximity to riparian systems like the Ganges, Brahmaputra, Irrawaddy and Indus Rivers. According to Hahn et al. [103] in a study of the roosting characteristics of flying foxes in Bangladesh an increase in colony size correlated positively with the distance to the nearest river (*p* = 0.03); a finding supported by studies in neighboring West Bengal, India [104]. One such region, the Greater Mekong Subregion has since 1970 lost 30% of its forest [105] with a predicted loss of 75% of its original forest and 42% of its mammal species by 2100 [106]. High disease transmission risk is distributed throughout a coastal corridor from southern Vietnam north through Hanoi, Nanning, Guangdong, and areas encompassing the global economic hubs of Hong Kong, Shenzhen, Guangzhou and the Pearl River Delta. The dangers associated with interspecies transmission events regionally are highlighted by the fact that China is home to about 50% of the world’s pig population [107]. Neighboring Vietnam, a country with a significant degree of overlap and disease transmission-risk, serves as the principal hub for pig exports regionally. Vietnam distributes pigs on trading routes through Thailand, Laos, Malaysia, Cambodia, Hong Kong and Singapore. Southeast Asia features multiple regional trading routes that are dictated by complicated supply and demand trends that vary considerably from country to county [108]. Cattle were the highest contributor to the human transmission model, a finding which implies that intensive agricultural practices are present in locations where spillover events have occurred. As reported by Chowdhury et al. [70] in a recent 2014 study, cattle and goats with NiVsG antibodies in Bangladesh were more likely to have had a history of eating fruit that had previously been partially consumed by nearby bat populations; these dropped fruits could have subsequently been contaminated with bat excreta or saliva. The serological response by the cattle in this study suggested a high likelihood of Henipavirus infection [70]. Cattle serve as an essential economic commodity for Bangladesh and Myanmar; both countries import up to two million head of cattle annually because of insufficient domestic production [108]. Malaysia is further dependent on the importation of live cattle to meet the rising domestic demand for meat products [108] (Figure 5).

The current study determined that land surface temperature (LST) and temperature seasonality was a strong predictor of presence in each model. Land surface temperature has been used as a proxy for numerous epidemiological studies on vector-borne diseases [109,110,111]. In predicting the distribution of the suspected bat reservoir of Ebola virus disease (EVD) in Africa and models of zoonotic transmission, Pigott [112] identified land surface temperature (LST) as a strong predictor of environmental suitability. According to the Global Animal Disease Intelligence Report (2016), land surface temperature is one of the factors/drivers influencing the dynamics of animal and zoonotic diseases globally [113]. In March, February and April 2016, significant above-average temperature anomalies (1.43 °C above 20th-century average) were observed in Papua New Guinea, northern and eastern Australia and southern Thailand [113]. Coupled with land surface temperature, seasonality is another catalyst for zoonotic and animal diseases [113]. Previous studies have pointed to seasonal changes in *Pteropus* behavioral patterns, especially during the dry season. For example, *Pteropus niger* frequently forages on cultivated fruits when their natural food sources are in short supply and are often observed in plantations, small holdings and home gardens [114]. A longitudinal study on the prevalence of NiV in Thailand among *Pteropus lylei* [12] found that the amount of virus and shedding bats fluctuated with both reproductive cycles and seasonality. Viral shedding was recorded in greater frequency during the first five months of the year. Additionally, the authors determined that the two viral strains: Malaysian and Bangladesh were detected in the urine of *P. lylei*, with the latter being dominant. This seasonality further corresponds with the dry season reproductive cycles of *P. giganteus* (November–April) in Bangladesh and India [12]. Seasonal fluctuations are additionally linked in the Russian Federation, Lithuania, Poland and Ukraine to epidemic waves of African swine fever (ASF). These variations are intertwined with the ecology of local wild boar populations [115].

**Figure 6:**
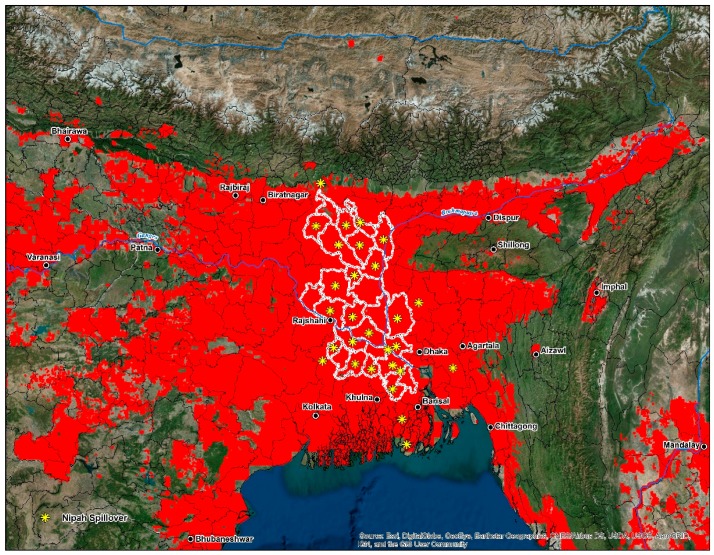
Intersecting cells of high-disease transmission risk superimposed on Bangladesh, eastern, and northeast India, and western Myanmar. The center of the map features the outline (white) of the ‘Nipah Belt’, an area of Bangladesh characterized by reoccurring outbreaks since 2001.

Recently a study on the temporal aspects of human cases found a correlation between yearly temperature differences and spillover events in Bangladesh from 2007–2013 [116]. Seasonality and the connection to date palm harvesting are common in human infections, with the majority occurring in the dry season between December and May [33]. Some mechanisms driving this trend include improved viral survivorship at colder temperatures and an increase in sap production during the winter months [116]. The cultivation of date palms has deep historical and cultural roots in Bangladesh and eastern India; it is a seasonal business for families living primarily in rural areas. Collecting sap is a critical component of the local economy and constitutes the livelihood of people during the winter when economic opportunities are lacking [117]. Date palm sap is collected early in the morning, distributed and consumed within hours before it ferments [116]. Multiple products are made from date palm sap; these include date palm wine, jaggery (*gur*), and sugar candy. Bangladeshi villages, where outbreaks have been documented, have one similarity in that a higher proportion of residents report consuming fresh date palm sap [116]. Similarly to Ebola virus disease (EVD), NiV causes high mortality rates in impoverished, rural communities [118,119]. Access to health care among these groups lacks considerably, even when these individuals face complications from severe illness [120]. Furthermore, the annual total per capita spending on health care nationwide in Bangladesh is estimated at $12 per person [121]. In Bangladesh (Figure 6), the majority of human cases are documented in the central and northwestern districts or the ‘Nipah Belt’. This area features land cover dominated by irrigated and rainfed croplands interspersed with grassland and forests. Villages found within the Nipah Belt feature high population densities and a high amount of forest fragmentation [103].

A variety of anthropogenic instigators propels the emergence of novel pathogens like NiV. Anthropogenic activities are a significant factor in bat-borne zoonosis transmission in human populations [5]. The continued fragmentation of sylvan landscapes through human-induced pressures has the potential of amplifying and increasing the likelihood of human–animal interactions [44,122]. The emergence of NiV is a clear example of amplification via agricultural encroachment through the establishment of monoculture plantations and the increased abundance of domestic animals [123]. Similar cases are documented with epidemics of Rift Valley fever (RVF) and Venezuelan equine encephalitis [124]. Preceding the 1998 outbreak was slash-and-burn deforestation for industrial plantations and pulpwood, followed by severe drought conditions exacerbated by the 1997–1998 El Nino southern oscillation (ENSO) [59]. Fragmentation propelled by urbanization leads to changes in connectivity among and between bat metapopulations, a phenomenon that has been identified in Australia as a driver of Hendra virus (HeV) infection in flying foxes [125]. These events result in a reduction of bat migration and exert pressure on the internal structure of bat populations facilitating spillover events [126]. Wilcox and Gubler [127] defined disturbances as contributing ‘to the natural disassembly of orderly natural communities’ through species ‘habitat simplification’ and ‘ecological release’. Investigations of the roosting behavior of *Pteropus giganteus* in Bangladesh indicate that with increasing population density and forest fragmentation came a propensity for the bats to roost in the remaining proximal tree canopy [44,103]. This behavior is typical in communities with previous zoonotic transmission to humans [112]. Landscape fragmentation and habitat loss have previously prompted bat colonies to seek alternative roosting sites on or near human dwellings [5,59]. Land cover change and deforestation in Bangladesh are attributed to poverty, land tenure rights, and unenforced forest management policy practices [128,129].

From 1970 to the mid-1990s, mango trees were planted near pig farms in Malaysia to increase agricultural output [14], a decision that ultimately intensified bat-pig interactions, the persistence of the virus, and human infection. The shift from vegetable-based diets to those with a higher intake of animal proteins is another factor [14]; zoonotic disease potential intensifies in proportion to the population of host animals and is commonly linked to an increased demand for meat products [130]. After the 1998 Malaysia outbreak, government restrictions were placed on fruit cultivation near poultry farms, resulting in policies that are now praised by public health officials [5]. Economically, the consequences of the initial NiV outbreak was devastating, along with the loss of human life, the Malaysian government estimates that 36,000 jobs and $350 million in revenue were lost during September 1998–May 1999 [7]. The economic impact of NiV in Bangladesh and India has yet to be assessed [131]. To account for the shift to commercialized monoculture, the incorporation of data representing tree plantations in Southeast Asia was a necessity. Although not contributing a high degree of variable importance to the human-transmission model (0.032) and being limited by the geographic extent of the dataset, this finding corroborates anecdotal evidence of potential risk factors for NiV spillover. Future modeling efforts would benefit from the inclusion of monoculture data for Bangladesh and Eastern India. With increases in international trade and commerce, the possibility for NiV pandemics in European, African, Eurasian, and East Asian economies is not out of the question. Biological interactions accelerating viral amplification include: (1) ecological changes related to economic development, land cover change, animal husbandry, climate change; (2) overpopulation; (3) international trade, commerce, and travel; (4) technological advancements in food processing; (5) microbe evolution; and (6) an overall decline in public health infrastructure [7,26,132]. Lederberg, Hamburg, and Smolinski [130] further stated that human development and large-scale social change are intimately associated with infectious diseases, and there is a need for research focused on ecological and social factors affecting disease emergence [130].

This study has several limitations and inherent challenges due to the multiple stages of analysis. The first is related to our choice of model variables, specifically the density of livestock. The initial outbreak in Malaysia was associated with the presence of pigs serving as an intermediate reservoir for the virus; however, Bangladesh is a majority Muslim country with a very low pig population density. This difference in livestock comparisons between nations explains the (0.085) variable importance of pigs in this study, due to the majority of human cases being documented in Bangladesh. Second, we are confident that the reported number of NiV human cases has been underreported throughout the study area. There are two reasons for this; one is that the available data on NiV in Bangladesh is biased towards those infections acquired during outbreaks only. The second possible explanation for underreported cases is that meningoencephalitis is a common cause of hospitalization in Bangladesh, it is plausible that NiV infection has been overlooked by medical professionals [33]. Raising awareness of the dangers of drinking raw date palm sap is an approach that may reduce the risk of zoonotic transmission. However, this may not be possible especially in rural areas. Third, ENMeval and BIOMOD2, as with any study that incorporates species distribution modeling (SDM), have inherent limitations. ENMeval features extended computation times due to the hundreds of replicate runs performed, a process intimately linked to the number of occurrence points and environmental variables in each analysis. Model evaluation methods like the area under the curve AUC (ROC) statistic is considered a relative standard of the geographic dissemination for a given study area and will discriminate occurrence from background localities. Background locations are treated as pseudo-absences for the evaluation and not the model fitting stage. The AUC has been criticized because it does reveal goodness-of-fit to provide information about the spatial distribution of model errors [133]. We recommend that the models presented here be interpreted with caution because they do not take into consideration the potential interaction between *Pteropus* bats and humans. We admit that even in geographies designated as high risk, it is difficult to quantify and predict with certainty zoonotic transmission. It must be emphasized that the presented maps do not enable the assessment of secondary transmission risk in human populations. In light of the reported results, the eco-epidemiology and ecology of NiV needs to be further explored.

## 5. Conclusions

Despite the caveats and limitations discussed above, the presented maps and associated results provide the best description of the geography of Nipah virus (NiV). This study offers an in-depth geographic and environmental analysis of Nipah virus (NiV) in South and Southeast Asia. The results of this analysis revealed that the emergence of NiV is complex and is interconnected with a plethora of cultural, geographic, ecological, and economic factors that facilitated its initial appearance and continued persistence. These complex interactions will be better understood through the continued development of interdisciplinary research collaboration between ecologists, medical doctors, veterinarians, epidemiologists, and geographers. Risk maps incorporating geospatial datasets are a valuable tool for the design of risk-based surveillance programs at multiple scales of analysis. Several key policy implications can be inferred from this study. First, the presented risk maps should significantly improve geographically targeted intervention strategies for policymakers attempting to maximize monetary resources and epidemic preparedness. Second, the presented maps can serve as an aid for our continued understanding of NiV host-virus ecology and the environmental spatial heterogeneity of disease transmission risk. Third, beyond the scope of public health entities at the local and national level, governments in geographic areas of high risk can utilize these maps to warn prospective economic stakeholders, local administrative officials, and travelers of the potential dangers associated with NiV. Recently, for the first time in India, NiV has been reported in Mangalore (Karnataka), Kozhikode and Malappuram districts (Kerala). According to local health officials, ten people who were exposed to NiV have died, and another three dozen people have been quarantined since the outbreak began on 21 May [134,135]. These new outbreak locations, which sit firmly in the high-risk portion of south India, have been included in the Supplementary Materials (S1) portion of this study.

## Figures and Tables

**Table 1:** Environmental variables.

Variable	Data Type	Resolution
Annual Mean Temperature	Environmental/continuous	2.5 arc-minutes
Mean Diurnal Range	Environmental/continuous	2.5 arc-minutes
Isothermality	Environmental/continuous	2.5 arc-minutes
Temperature Seasonality	Environmental/continuous	2.5 arc-minutes
Max. Temperature of Warmest Month	Environmental/continuous	2.5 arc-minutes
Min. Temperature of Coldest Month	Environmental/continuous	2.5 arc-minutes
Temperature Annual Range	Environmental/continuous	2.5 arc-minutes
Mean Temperature of Wettest Quarter	Environmental/continuous	2.5 arc-minutes
Mean Temperature of Driest Quarter	Environmental/continuous	2.5 arc-minutes
Mean Temperature of Warmest Quarter	Environmental/continuous	2.5 arc-minutes
Mean Temperature of Coldest Quarter	Environmental/continuous	2.5 arc-minutes
Annual Precipitation	Environmental/continuous	2.5 arc-minutes
Precipitation of Wettest Month	Environmental/continuous	2.5 arc-minutes
Precipitation of Driest Month	Environmental/continuous	2.5 arc-minutes
Precipitation Seasonality	Environmental/continuous	2.5 arc-minutes
Precipitation of Wettest Quarter	Environmental/continuous	2.5 arc-minutes
Precipitation of Driest Quarter	Environmental/continuous	2.5 arc-minutes
Precipitation of Warmest Quarter	Environmental/continuous	2.5 arc-minutes
Precipitation of Coldest Quarter	Environmental/continuous	2.5 arc-minutes
Evergreen Broadleaf	Landscape/categorical	1-km
Croplands	Landscape/categorical	1-km
Gross Canopy Loss (2000–2016)	Human environment/categorical	30-m
Elevation (SRTM)	Landscape/continuous	1-km
Mean MODIS EVI (2001–2012)	Landscape/continuous	1-km
Mean Value 8-day MODIS day-time Land Surface Temperature (LST) (2011–2012)	Landscape/continuous	1-km
Tree Plantations (Southeast Asia)	Landscape/categorical	1-km
Mixed Forests	Landscape/categorical	1-km
Mosaic Cropland/Vegetation	Landscape/categorical	1-km
Woody Savannas	Landscape/categorical	1-km
Human Population Density (2015)	Human environment/continuous	1-km
Cattle Density	Landscape/categorical	1-km
Goat Density	Landscape/categorical	1-km
Sheep Density	Landscape/categorical	1-km
Pig Density	Landscape/categorical	1-km

**Table 2:** Model evaluation statistics (average)—reservoir model.

Model	ROC	KAPPA	TSS
GLM	0.873	0.606	0.627
GBM	0.886	0.654	0.663
FDA	0.867	0.616	0.616
MARS	0.868	0.607	0.621
RF	0.897	0.661	0.681

**Table 3:** Top model contributors—reservoir model.

Variable	Contribution	Sample Averages
Population Density	0.286	1357 per cell
Mean Temperature of the Driest Quarter	0.252	23 °C
Precipitation of the Warmest Quarter	0.143	1124 mm
Land Surface Temperature (LST)	0.124	-
Temperature Seasonality	0.117	30 °C
Elevation (SRTM)	0.074	709 m
EVI MODIS (2001–2012)	0.060	-
Mean Temperature of Warmest Quarter	0.039	28°C
Mosaic Vegetation	0.036	-
Cattle Density	0.028	24.85
Pig Density	0.025	18.84

**Table 4:** Model evaluation statistics—human transmission model.

Model	ROC	KAPPA	TSS
GLM	0.862	0.615	0.629
GBM	0.917	0.752	0.772
FDA	0.857	0.611	0.623
MARS	0.902	0.729	0.734
RF	0.770	0.747	0.789
Maxent. Phillips	0.770	0.526	0.517
Maxent. Tsuruoka	0.878	0.627	0.675

**Table 5:** Top model contributors (average)—human transmission model.

Variable	Contribution	Sample Averages
Cattle Density	0.509	45.5
Temperature Seasonality	0.201	40.2 °C
Elevation (SRTM)	0.115	857.45 m
Land Surface Temperature (LST)	0.105	-
Population Density	0.103	348.71 per cell
Mean Temperature of the Driest Quarter	0.097	21 °C
Mean Temperature of the Warmest Quarter	0.088	28 °C
Sheep Density	0.06	16.7
Pig Density	0.06	16
Mosaic Vegetation	0.051	-
Tree Plantations	0.032	-
Precipitation of the Warmest Quarter	0.029	980 mm

**Table 6:** Land area designated as ‘high risk’.

Country	Area km^2^
Afghanistan	15.29
Australia	1258
Bangladesh	122,951
Brunei	2929
Cambodia	96,421
China	68,223
Hong Kong	263
India	549,588
Indonesia	1,107,777
Laos	19,640
Macau	4.88
Malaysia	206,031
Myanmar	90,354
Nepal	8092
Pakistan	18,626
Papua New Guinea	17,540
Philippines	233,641
Singapore	0.97
Sri Lanka	51,430
Taiwan	8187
Thailand	192,403
Timor-Leste	23,554
Vietnam	148,584

## Supplementary Materials

The following are available online at http://www.mdpi.com/2414-6366/3/2/57/s1. Map S1: Nipah virus disease transmission risk in Bangladesh and India with updated human case locations from May 2018. Map S2: Nipah virus disease transmission risk in Southeast Asia. Map S3: Nipah virus disease transmission risk in Indonesia.
