# Supplementary figures and images for: Mapping Disease Transmission Risk of Nipah Virus in South and Southeast Asia
Source: Trop Med Infect Dis. 2018 May 30;3(2):57. doi: 10.3390/tropicalmed3020057 (PMC6073609; doi:10.3390/tropicalmed3020057)

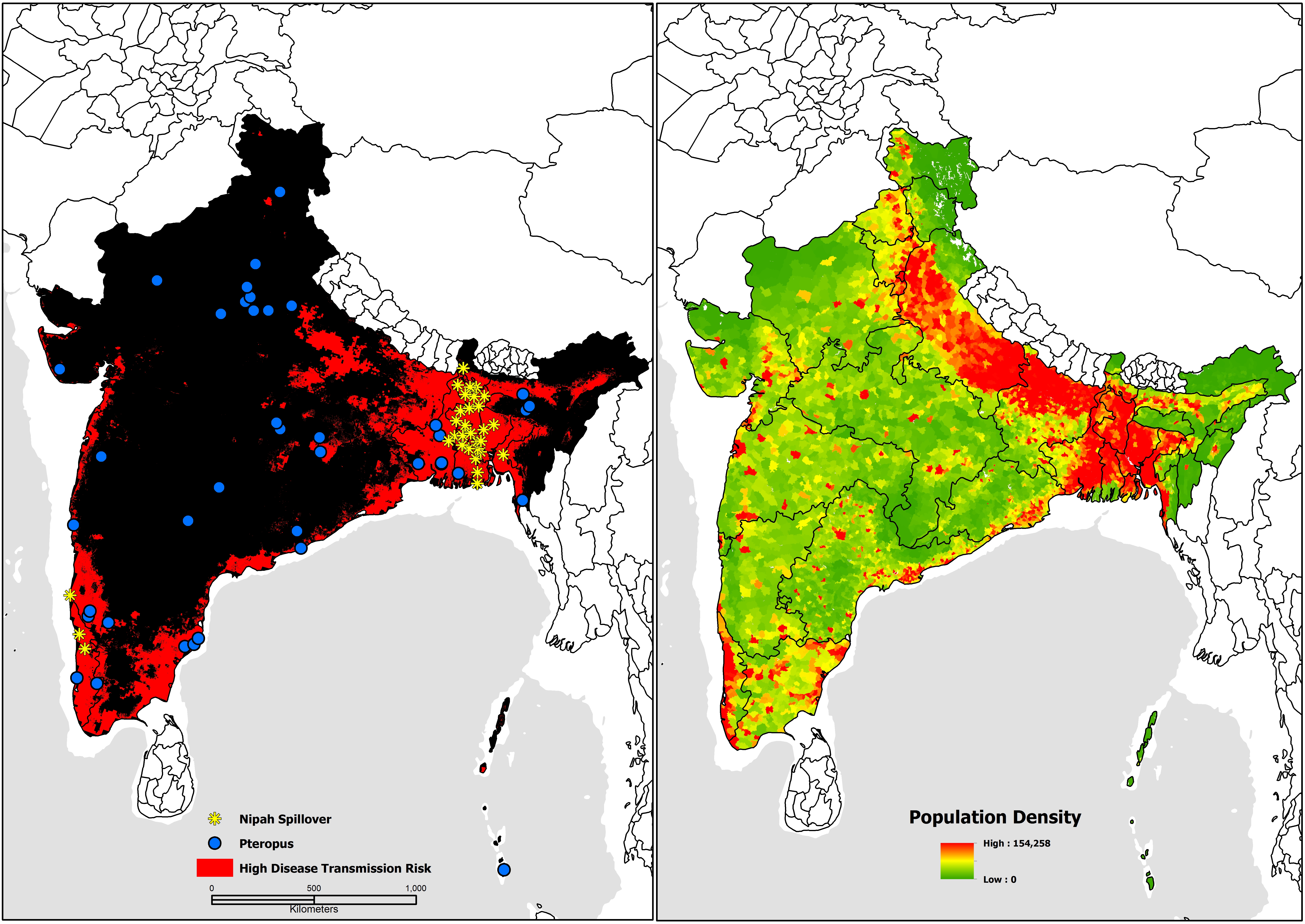

Supplement: Supplementary file 1 [file tropicalmed-03-00057-s001.zip › S1.jpg]

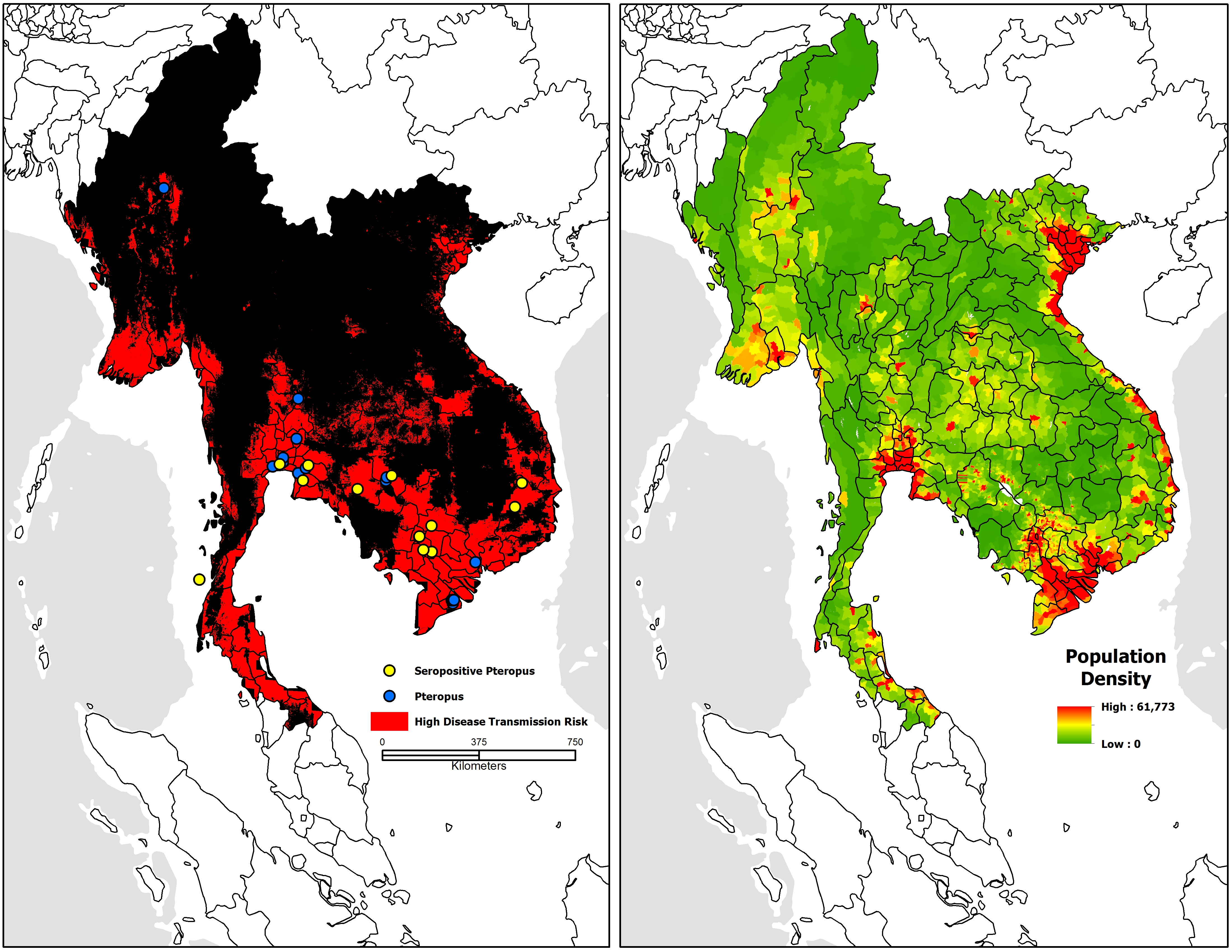

Supplement: Supplementary file 1 [file tropicalmed-03-00057-s001.zip › S2.jpg]

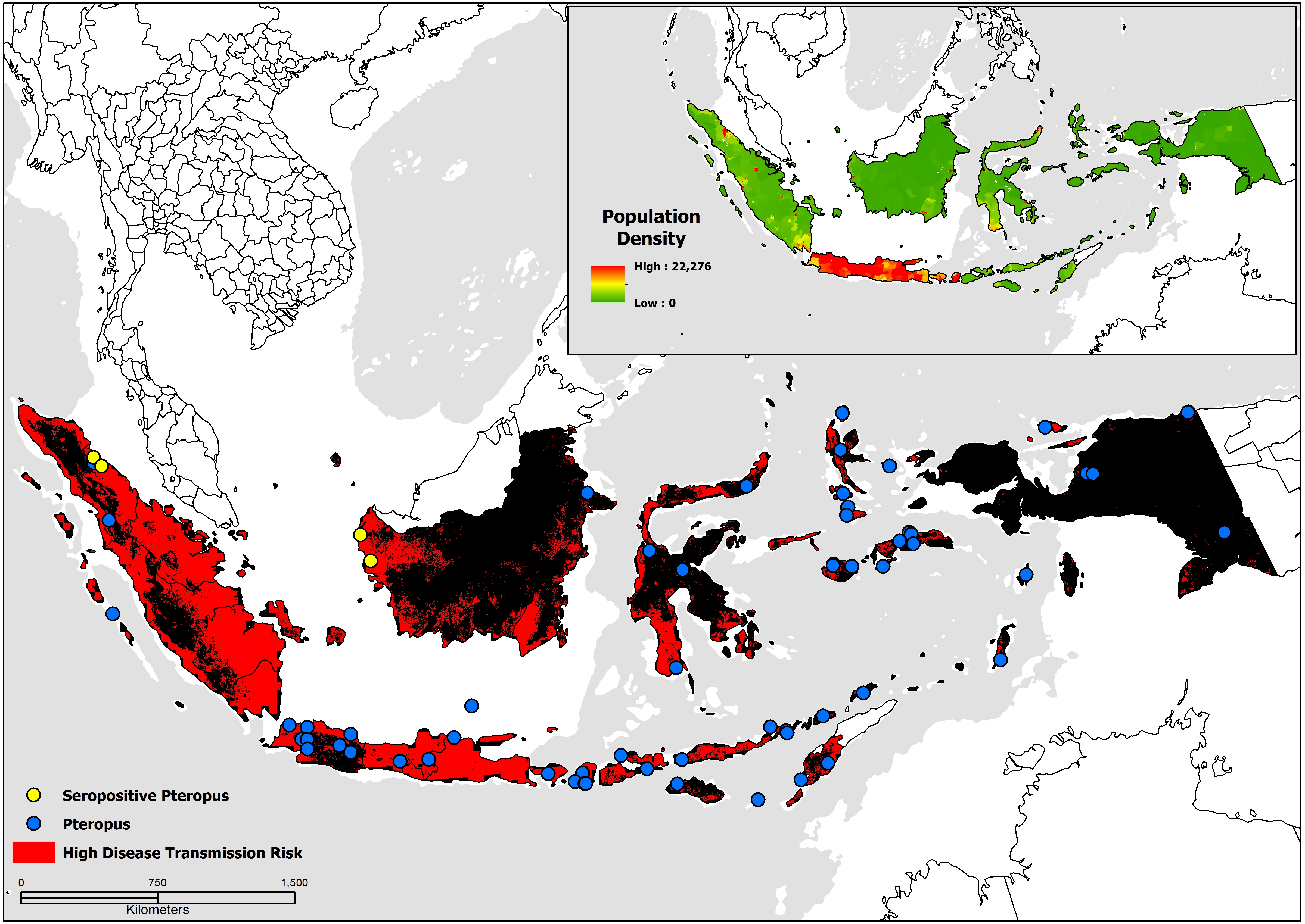

Supplement: Supplementary file 1 [file tropicalmed-03-00057-s001.zip › S3.jpg]
